# ImaGene: a web-based software platform for tumor radiogenomic evaluation and reporting

**DOI:** 10.1093/bioadv/vbac079

**Published:** 2022-11-10

**Authors:** Shrey S Sukhadia, Aayush Tyagi, Vivek Venkataraman, Pritam Mukherjee, Pratosh Prasad, Olivier Gevaert, Shivashankar H Nagaraj

**Affiliations:** Centre for Genomics and Personalised Health, Queensland University of Technology, Brisbane, QLD 4000, Australia; Translational Research Institute, Brisbane, QLD 4000, Australia; Yardi School of Artificial Intelligence, Indian Institute of Technology, New Delhi 110016, India; Centre for Genomics and Personalised Health, Queensland University of Technology, Brisbane, QLD 4000, Australia; Translational Research Institute, Brisbane, QLD 4000, Australia; Stanford Center for Biomedical Informatics Research, Department of Medicine and Biomedical Data Science, Stanford University, Stanford, CA 94305-5101, USA; Department of Electrical Communication Engineering, Indian Institute of Science, Bangalore 560012, India; Stanford Center for Biomedical Informatics Research, Department of Medicine and Biomedical Data Science, Stanford University, Stanford, CA 94305-5101, USA; Centre for Genomics and Personalised Health, Queensland University of Technology, Brisbane, QLD 4000, Australia; Translational Research Institute, Brisbane, QLD 4000, Australia

## Abstract

**Summary:**

Radiographic imaging techniques provide insight into the imaging features of tumor regions of interest, while immunohistochemistry and sequencing techniques performed on biopsy samples yield omics data. Relationships between tumor genotype and phenotype can be identified from these data through traditional correlation analyses and artificial intelligence (AI) models. However, the radiogenomics community lacks a unified software platform with which to conduct such analyses in a reproducible manner. To address this gap, we developed ImaGene, a web-based platform that takes tumor omics and imaging datasets as inputs, performs correlation analysis between them, and constructs AI models. ImaGene has several modifiable configuration parameters and produces a report displaying model diagnostics. To demonstrate the utility of ImaGene, we utilized data for invasive breast carcinoma (IBC) and head and neck squamous cell carcinoma (HNSCC) and identified potential associations between imaging features and nine genes (WT1, LGI3, SP7, DSG1, ORM1, CLDN10, CST1, SMTNL2, and SLC22A31) for IBC and eight genes (NR0B1, PLA2G2A, MAL, CLDN16, PRDM14, VRTN, LRRN1, and MECOM) for HNSCC. ImaGene has the potential to become a standard platform for radiogenomic tumor analyses due to its ease of use, flexibility, and reproducibility, playing a central role in the establishment of an emerging radiogenomic knowledge base.

**Availability and implementation:**

www.ImaGene.pgxguide.org, https://github.com/skr1/Imagene.git.

**Supplementary information:**

Supplementary data are available at https://github.com/skr1/Imagene.git.

## 1 Introduction

Diagnostic imaging techniques are routinely used in clinics and laboratories for the identification of tumor severity and progression (Bodalal *et al.*, 2019). Common diagnostic techniques include computed tomography (CT), magnetic resonance imaging (MRI) and positron emission tomography (PET). These techniques yield high-quality digital images for tumor assessment that are also suitable for building databases that curate patient data for the purposes of research reproducibility and reuse (Diaz *et al.*, 2021; Freymann *et al.*, 2012; Gillies *et al.*, 2016; Prior *et al.*, 2017).

When used to assess tumors, diagnostic imaging techniques produce images that are examined by radiologists to segment (or select) tumor regions of interest (ROIs) that are believed to represent core sections of tumor on images. Measurements such as spatial, volumetric, textural and intensity-based are extracted from image-based ROIs and analyzed using several techniques in correlation analysis and artificial intelligence (AI) (Pfaehler *et al.*, 2019; van Griethuysen *et al.*, 2017). In parallel, a portion of the corresponding tissue material is biopsied from patient’s body and subjected to histopathological examination (Lee *et al.*, 2013; Rice *et al.*, 2017) and often also omics-based assessment (González-Reymúndez and Vázquez, 2020; Liu *et al.*, 2018; Nasrallah *et al.*, 2019; Peng *et al.*, 2015; Wang *et al.*, 2018). Omics-based investigations yield information such as gene expression, copy number variations (CNVs) or other structural variants (SVs), single-nucleotide variations (SNVs) and DNA methylation scores for genes that regulate biological processes within the biopsied tissue material (Anker *et al.*, 2015; Balassiano *et al.*, 2011; Chen *et al.*, 2013; Park *et al.*, 2010).

Despite the widespread use of tumor biopsies as a means of establishing important diagnostic information pertaining to specific ROIs, several studies have reported an underestimation of adverse pathologies in up to 23% of the samples due to spatial sampling bias (Aihara *et al.*, 1994; Kvale, 2009; Martin-Gonzalez *et al.*, 2020; Siddiqui *et al.*, 2015; Smith *et al.*, 2019). This may be attributed to the spatial heterogeneity of morphological growth patterns (Aihara *et al.*, 1994; Bosaily and El-Shater, 2016; Boutros *et al.*, 2015) as well as genetic heterogeneity within the cancerous lesions and may result in the underestimation of tumor severity, leading to an increase in the potential for tumor-related adverse events in these patients (Aihara *et al.*, 1994; Incoronato *et al.*, 2020; Martin-Gonzalez *et al.*, 2020; Sottoriva *et al.*, 2013). Therefore, combining imaging information of tumors with their omics profile could explain the tumor heterogeneity better than just with imaging alone. One such method would be to correlate data from image-based tests and omics-based investigations to improve the quality of diagnosis (Bakr *et al.*, 2018; Gevaert *et al.*, 2012, 2014; Lo Gullo *et al.*, 2020; Martin-Gonzalez *et al.*, 2020; Zhou *et al.*, 2018) and to provide more reliable tumor ROIs that can be used for tissue sampling when performing biopsies (Gillies *et al.*, 2016; Martin-Gonzalez *et al.*, 2020). Furthermore, AI models built with flexibility in performing prior filtering of features using statistical correlation analysis may allude crucial associations that may lead to improved tissue biopsies in patients (Ashraf *et al.*, 2014; Bodalal *et al.*, 2019; Chitalia *et al.*, 2020). Such models may also reduce or obviate the need for tissue biopsy for tumor assessment in near future (Bodalal *et al.*, 2019).

The field of imaging genomics or radiogenomics focuses on finding associations between radiomic characteristics of tissue ROIs and molecular characteristics such as genomic, transcriptomic and proteomic profiles of tumor cells. Using data from The Cancer Imaging Archive (TCIA), a group recently conducted a locoregional study using MRI images of glioblastoma tissues by generating heat maps corresponding to both core and boundary regions of specific tumor ROIs, revealing substantial genetic heterogeneity (Depeursinge *et al.*, 2018). The gene expression profiles of each of the sampled regions were correlated with known information regarding the genes involved in multiple pathways potentially leading to oncogenesis (Depeursinge *et al.*, 2018). Another recent study investigated gene expression profiles of pancreatic ductal adenocarcinoma (PDAC) ROIs using CT images by comparing the radiomic features of these ROIs with their genotypes and stromal content (Attiyeh *et al.*, 2019). This investigation yielded a radiogenomic model that provided important information regarding the number of altered genes in the ROIs and SMAD4 gene expression status in association with radiomic features. Stromal content was also correlated with radiomic and genomic features of the ROIs, thereby indicating that stromal information can guide decision-making for PDAC samples (Attiyeh *et al.*, 2019).

Currently, the field of tumor diagnostics relies on the biopsy of tissue samples followed by molecular analyses to identify the genomic characteristics of a given tumor (Kamel and Al-Amodi, 2017). This approach is limited to providing information restricted to the biopsied tissues and does not reveal the heterogeneity of the tumor or the underlying changes that take place at the cellular and tissue levels (Kamel and Al-Amodi, 2017). The assessment of radiomic features derived from radiographic scans such as CT and MRI can provide a noninvasive means of gauging overall survival of tumor patients (Shukla *et al.*, 2017). Radiomic features, in combination with clinical and genomic information, can aid clinicians in deciding the best course of therapy for these patients (Shukla *et al.*, 2017). To overcome the limitations of histopathological analysis and to facilitate the acquisition of in-depth insights into tumor molecular characteristics, there is a pressing need for the development of tools that can perform advanced statistical and correlational analyses using data derived from both histological as well as diagnostic imaging approaches (Mobadersany *et al.*, 2018).

Radiogenomic investigations typically use a combination of machine learning and statistical methods to detect correlations between the radiomic and genomic features of tumor regions. These methods are unique for each study and most studies do not provide the details of the codes and parameters used for these investigations, thereby limiting their reproducibility (Brito *et al.*, 2020; Colen*et al.*, 2014). In addition, they do not offer users with enough algorithmic flexibility, for example, using AI modeling with or without prior correlations filtering of the features, the approaches practiced commonly in the field so far (Ashraf *et al.*, 2014; Chitalia *et al.*, 2020; Trivizakis *et al.*, 2020). There is currently a need for the development of a sophisticated, user-friendly, web-based software platform that can perform both correlation analysis and modeling operations using AI methods and provide trained models that could be used for rigorous testing of associations of imaging features with the omics profiles of tumor ROIs (Bodalal *et al.*, 2019; Colen *et al.*, 2014). There is also a need for a transparent algorithm, the parameters of which can be substantially exposed and altered to obtain comprehensive and self-explanatory reports that can be used for radiogenomics research and hopefully cancer diagnosis post sufficient validations through an unified software platform.

At present, there are several software tools available that can detect associations between imaging characteristics and gene regulatory networks in tissue samples, including Imaging-Amaretto and Imaging-Community Amaretto (Gevaert *et al.*, 2020). However, these tools neither have a sophisticated, user-friendly, web-based platform that can allow for the straightforward manipulation of experimental parameters, nor do they provide comprehensive reports that describe statistical correlations between imaging and omics features using clustered heat maps, and the AI-based prediction and/or classification of labeled data along with the metrices such as root mean square error (RMSE):standard deviation (STDEV) ratio, *R*^2^ and area under the receiver operating curve (AUC) that explain the performance of models in predicting and/or classifying either omics or imaging data from either imaging or omics data, respectively (Gevaert *et al.*, 2020). In addition, users require a flexibility to select from a variety of AI model types (mainly regression based popular in radiogenomic domain; Attiyeh *et al.*, 2019; Depeursinge *et al.*, 2018) for training and testing, and consequently postanalyzing or comparing results using quality control metrices such as RMSE, STDEV, *R*^2^ and AUC to arrive at reliable imaging-omics associations (Gevaert *et al.*, 2020). Multiomics Statistical Approaches is another tool used for radiogenomic studies that provides correlation heat maps and principal component analysis plots (Zanfardino *et al.*, 2021). However, it lacks a feature prediction ability imparted by AI-based methods (Zanfardino *et al.*, 2021). All these tools also fail to adhere to the Findability, Accessibility, Interoperability, and Reusability (FAIR) principles that allow users to store, track and analyze their data through both individual steps and the entire experiment (Wilkinson *et al.*, 2016).

To address this need for a universal platform that can conduct correlation analysis between imaging and omics-based features of tumor ROIs and build AI models based on or off such correlated features, we developed ImaGene. ImaGene is a web-based software platform that integrates statistical and AI techniques to facilitate tumor radiogenomic analyses. ImaGene facilitates systematic radiogenomic analysis using various statistical and AI parameters that allow users to configure their experiments and perform appropriate iterations thereof. The end result of this platform is an HTML document that describes how the experiment was executed, the parameters that were used, and the resulting associations in the form of correlation plots and performance metrics of AI models including RMSE and the RMSE-to-STDEV ratio. The AI piece consists of regression models such as linear, regularized regression (LASSO and elastic net along with their respective multi-task versions) and decision trees (DT). It further conducts classification of predicted labels at various decision thresholds (dts) yielding AUC values at such thresholds. Through these outputs, ImaGene allows users to perform their radiogenomic experiments more intuitively and helps them establish unambiguous conclusions that can highlight data-driven directions for future research. ImaGene provides an integrative platform that can be used with data from both histological as well as diagnostic imaging to reveal tumor-related spatial and temporal details that cannot be established through histopathology alone.

## 2 Methods

### 2.1 Features of ImaGene

**Fig. 1.**
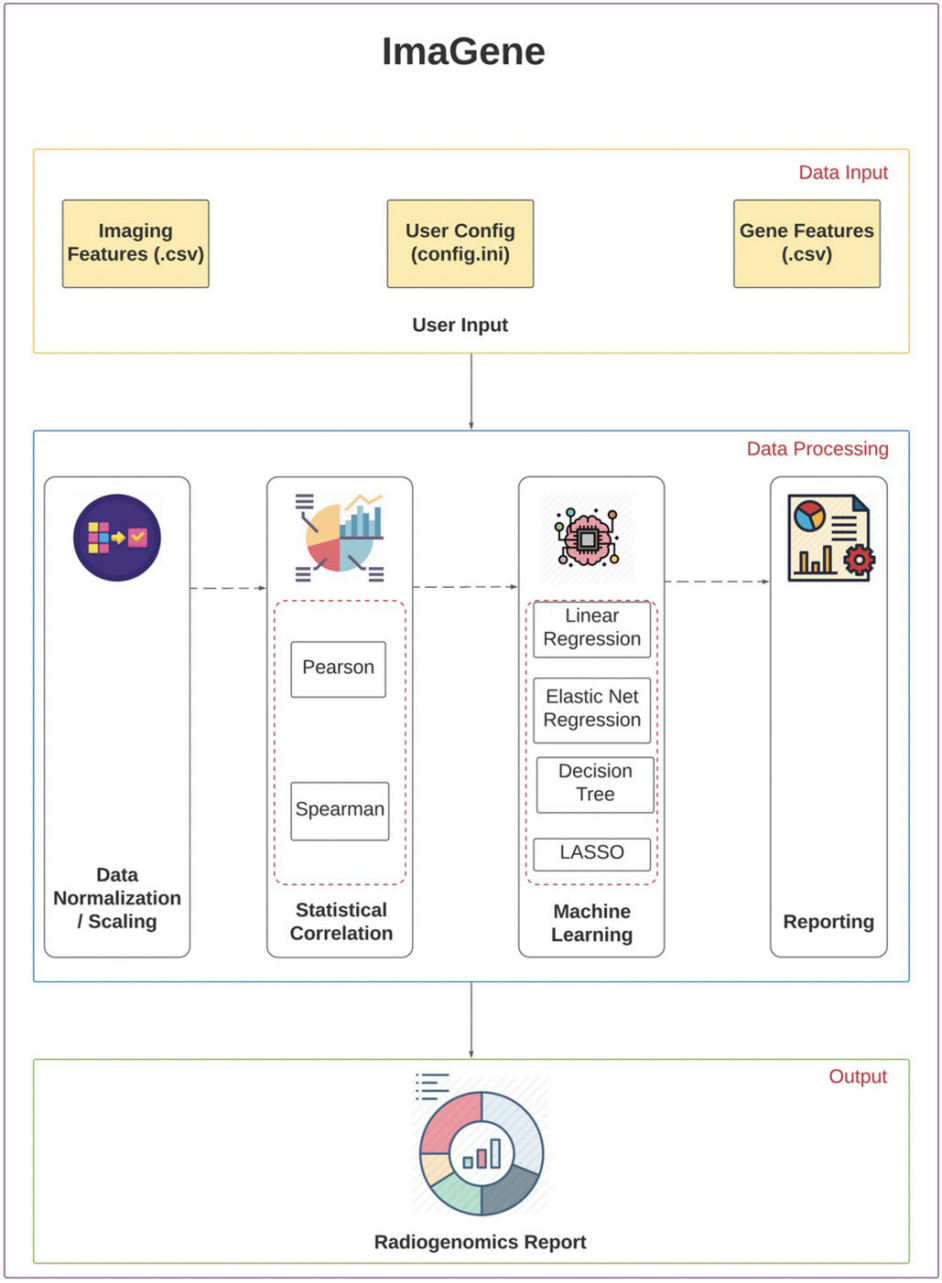
ImaGene’s Schema depicting its components

ImaGene has been deployed as a Graphical User Interface (GUI) on the Amazon Web Service (AWS) servers of Queensland University of Technology (QUT) and is also available as an open-access website ‘www.ImaGene.pgxguide.org’ (Fig. 3). Users can register to the website for a free account. Alternatively, users can download ImaGene from GitHub (https://github.com/skr1/Imagene.git) and operate it on a Linux Operating System (OS) using a Command-Line Interface (CLI). Once downloaded, users can set various configuration parameters available on the platform and run their experiments (Fig. 3). ImaGene comprises of four modules: (i) data preprocessing, (ii) correlation analysis, (iii) machine learning (ML) and (iv) reporting (Fig. 1). The code for the software has been written in Python, and it utilizes several libraries such as scikit-learn (Pedregosa *et al.*, 2011), matplotlib, seaborn and importr along with custom functions written to follow a systematic approach to analyze and pin point meaningful associations between imaging and omics features.

For analytical operations, ImaGene requires two input files, each in Comma-Separated Value (CSV) format—one containing imaging features (and their measurements) and another containing omics features for a set of tumor samples. The imaging features can be acquired from feature extraction software such as PyRadiomics, LIFEx or RaCaT by processing the tumor images using the respective segmentation labels (van Griethuysen *et al.*, 2017; Koçak *et al.*, 2019; Pfaehler *et al.*, 2019). The omics features can be acquired from studies conducted on tumor ROIs in biopsy samples processed in pathological laboratories and may consist of data pertaining to gene expression, SV (including CNV), SNV or DNA methylation scores.

Once the imaging and omics feature files are uploaded in the software, the names and order of samples therein are matched. Following this, the feature files are analyzed for different configuration parameters based on values specified by the user in the data preprocessing module (Table 1 and Fig. 1). The imaging and omics features undergo normalization or scaling based on the normalization method set by the user such as Standard Scaler, Max Absolute Scaler and MinMax Scaler (Table 1). Normalization occurs independently for training and test datasets using the same normalization technique to prevent the leak of test data into train data.

**Table 1.** Configuration parameters for ImaGene

Configuration parameters	Values
Correlation method	Default: Spearman (options: Pearson)
Correlation coefficient threshold	Default: 0.5 (Set ‘−1.0’ to disable correlation-coefficient-threshold-based filtering of features)
*P*-Value correction method	Default: BH (options: Holm, Hochberg, Hommel, Bonferroni, BH, BY and FDR)
Data feature type	Default: Imaging (option: Gene)
Label feature type	Gene (option: Imaging)
Train size	0.9 (flexible)
Test size	0.1 (flexible)
Normalization method	Default: Stand_scaler (options: min_max, Stand_scaler and MaxAbsScaler)
Mode	Default: Train (options: Train, Validate and Predict)
Model type	Default: Decision Tree (options: Linear Regression, Linear Model aka Elastic-Net, LASSO, Multi-Task LASSO and Multi-Task Elastic Net)
Grid search	default: False (options: True and False)
Cross-validation splitter	cv: 2 (flexible)
<Section for other model parameters for each model type>	Accepts as many model parameters as possible per scikit-learn library. Can be empty
<Section for Grid Search parameters for each model type>	Accepts as many grid search parameters as possible per scikit-learn library. Can be empty

The data is then transferred to the correlation analysis module where either Pearson or Spearman test is conducted for imaging and omics features per user’s selection of the correlation method (Table 1). The outcome of the correlation test is a subset of imaging and omics features that are highly correlated with one another (correlation coefficient >0.5). The correlation threshold configuration parameter allows users to specify this minimum correlation coefficient threshold (Table 1). For strong correlations found in the imaging and omics feature files, the default Benjamin–Hochberg (BH) *P*-value correction method (Table 1) is used to measure and adjust the *P*-values for false discovery rates. Features having significant correlations are presented as hierarchically clustered heat maps depicting three types of relationships between imaging and omics features—(i) two-way univariate, (ii) univariate to multivariate and (iii) two-way multivariate. These features (*P*-adjust < 0.05) are then transferred to the ML module for further processing. Setting the correlation threshold to −1.0 disables correlation threshold-based filtering of imaging and omics features and transfers all features to the ML module.

In the ML module, the statistically correlated features are used to construct a definitive ML model based on the model type specified in the parameter settings (Table 1). Currently, the model types available in the platform are linear, regularized regression (LASSO and elastic net along with their respective multi-task versions) and DT. Users may specify their preferred model parameters (Table 1) depending on the options available in the scikit-learn library (Pedregosa *et al.*, 2011). Alternatively, if users are unsure of these parameters, they may leave this section empty or specify only a subset of parameters, in which case, default values for unspecified parameters will be used (Fig. 3b). Users also have the flexibility to specify omics and imaging features as either data, which is the independent variable set (*X*) where, {$X=\{x_{1},x_{2}\ldots x_{n}\}\}\;$or label, which is the dependent variable set (*Y*) where {$Y=\{y_{1},y_{2}\ldots y_{n}\}$} and which is predicted based on the independent variable set (*X*). ImaGene also provides users with an option to specify grid parameters for their experiments based on the options available in the scikit-learn library, which can be achieved by setting the ‘grid search’ parameter to ‘True’ (Fig. 3b), and by either mentioning the list of grid search parameters in the ‘Model Parameters’ section (Fig. 3b) or by using the default options. Specifying grid search parameters allows users to train models using different settings for model parameters, finalizing a model with the best performing parameter settings.

The ML module uses either user-defined settings or default settings to construct an AI model that is trained using a training dataset. Following this, a *K*-fold cross-validation is performed to reduce data overfitting based on the value specified in the ‘Cross-Validation Splitter’ parameter (cv, default value = 2) (Table 1). Users can also specify the fraction of the total number of test samples that should be used for testing the model by entering their desired value in the ‘Test Size’ parameter (default value = 0.1) (Table 1 and Fig. 3b). Based on this setting, the test dataset is used for testing the model.

The test dataset is scored by calculating the negative mean square error (MSE) and the RMSE. RMSE is calculated for each label (*y*) in the dataset, and the ratio of RMSE ‘RMSE(y)’ and standard deviation of the originally observed values ‘STDEV(y)’ (RMSE:STDEV) represents the prediction error of the model for each label as a function of the standard error (standard deviation) in the distribution of the observed label values (*y*_observed_). Furthermore, users can utilize the identity (1) below depicting the relationship between *R*^2^ and RMSE:STDEV ratio (v) to compute the *R*^2^. The *R*^2^ along with *v* provides users with two metrics that aid in assessing the reliability of various regression models in the context of residual variance for *y*_predict_ (i.e. SSL) and the total variance that exists in *y*_observed_ (i.e. SST).

$$v=\frac{\text{RMSE}(y)}{\text{std}(y)}=\sqrt{\frac{\text{MSE}(y)}{\text{Var}(y)}}=\sqrt{\left(\frac{\frac{1}{n}{\left\|y-\hat{y}\right\|}^{2}}{\frac{1}{n}{\left\|y-\bar{y}\right\|}^{2}}\right)}=\sqrt{\frac{\text{SSL}}{\text{SST}}}$$

$$\text{By}\;\text{definition},\;R^{2}=1-\frac{\text{SSL}}{\text{SST}}$$

Therefore, from the above, it is derived that

(1) $$R^{2}=1-{\left(v\right)}^{2}$$

In addition, ImaGene also calculates *R*^2^ using the r2-score module in scikit-learn library (Pedregosa *et al.*, 2011). Interestingly, these *R*^2^s were found to match the *R*^2^s calculated using the identity (1) (Tables 2 and 3).

**Table 2.** Significant and explainable gene predictions at different normalized FPKM thresholds using various radiogenomic models in the invasive breast carcinoma case study

Model	Genes	Normalized FPKM thresholds	AUC	*R* ^2^	*R* ^2^ ^a^	AUC *P*-value	*R* ^2^ *P*-value	Biological significance
DT	WT1	[0.5, 0.9]	1.0	0.48	0.48	0	0	Yes
	LGI3	[0.6, 0.9]	0.94	0.36	0.36	0	0	Yes
	SP7	[0.4, 0.9]	1.0	0.33	0.33	0	0	Yes
MTEN	SLC22A31	[0.3, 0.9]	1.0	0.75	0.75	0	0	Yes
	SMTNL2	[0.3, 0.9]	1.0	0.33	0.33	0	0	Yes
	DSG1	[0.4, 0.7]	0.94	0.35	0.35	0	0	Yes
MTL	ORM1	[0.7, 0.9]	1.0	0.33	0.33	0	0	Yes
	CLDN10	0.9	1.0	0.37	0.37	0	0	Yes
	CST1	[0.6, 0.9]	1.0	0.28	0.28	0	0	Yes
LR	SLC22A31	[0.3, 0.9]	1.0	0.72	0.72	0	0	Yes

a Calculated using identity (1).

**Table 3.** Significant and explainable gene predictions at different FPKM thresholds using various radiogenomic models in the HNSCC case study

Model	Genes	Normalized FPKM thresholds	AUC	*R* ^2^	*R* ^2^ ^a^	AUC *P*-value	*R* ^2^ *P*-value	Biological significance
DT	NR0B1	[0.3, 0.9]	1.0	0.99	0.99	0	0	Yes
	PLA2G2A	0.9	1.0	0.4	0.4	0	0	Yes
LR (corr_threshold = 0.7, *P-*adjust <0.05)	MAL	[0.8, 0.9]	0.9	0.41	0.41	0	0	Yes
MTL	CLDN16	0.9	0.93	0.3	0.3	0	0	Yes
MTEN	PRDM14	[0.3, 0.9]	1.0	0.3	0.3	0	0	Yes
	VRTN	0.2 and [0.6–0.8]; 0.9	0.93; 1.0	0.46	0.46	0	0	Yes
	LRRN1	[0.6–0.9]	0.93	0.32	0.32	0	0	Yes
	MECOM	[0.6, 0.9]	1.0	0.32	0.32	0	0	Yes

a Calculated using identity (1).

In order to evaluate the performance of a regression model when used to classify omics labels (such as genes) into groups of their high and low measures (for instance, high and low normalized gene expressions which are in continuous form), such measures are first binarized for *y*_observed_ at various dts (aka normalized gene expression cutoffs) for each and every gene label (*y*). Thus, if a normalized expression value belongs to a range of [0,1] increasing in steps of 0.1 (i.e. dt={0.1, 0.2…1.0}), and if the value of the dt is set to 0.5 (as an example), all the expression values (*y*_observed_) falling below 0.5 will be set to 0 and the ones rising above 0.5 will be set to 1 for every gene label (*y*). The *y*_predict_ however would be retained as they are, i.e. in continuous form. Such a binarization of *y*_observed_ allows ImaGene to calculate AUCs for each label (gene) at various measurement cutoffs (i.e. dts) ultimately aiding users deem the best dt (or range of dts) at which a model is able to classify a label (gene) as high or low measured (i.e. expressed) for the given spectrum of imaging features (Depeursinge *et al.*, 2018). A plot of the AUC values versus dts is generated for all gene labels providing users with a visualized overview (along with the respective output data files) indicative of a regression model’s classification performance. This is very beneficial from biological perspective as users can determine expression cutoffs for a gene at which a regression model in radiogenomics can be relied upon for predicting that gene as high or low expressed using imaging features from a tumor ROI on the radiographic image.

ImaGene performs permutation tests for the genes predicted at AUC > 0.9 and *R*^2^ > 0.25 during the test phase such that their significance can be estimated. The results from these tests are text files labeled as ‘validation permuts’, so that users can calculate *P*-values. The *P*-values can be calculated as no. of times AUC_permut_ ≥ AUC_test_ and *R*^2^_permut_ ≥ *R*^2^_test_ for AUC and *R*^2^ (respectively) for each gene called with AUC_test_ > 0.9 and *R*^2^_test_ > 0.25 during the test phase. These thresholds were kept high to eliminate the permutation runs (tests) on genes showing low *R*^2^_test_ and AUC_test_ values and to focus on confidently predicted genes only. This also helps keep the runtime of ImaGene optimal. These two features contribute to ImaGene’s robustness and reliability. In addition, ImaGene reports feature importances results as text files depicting how each radiomic feature weighted in prediction of a gene. These weights are either in the form of feature importances called from a Decision Tree Regressor or are model coefficients in case of other regression models, both calculated using respective modules in scikit-learn library (Pedregosa *et al.*, 2011).

**Fig. 2.**
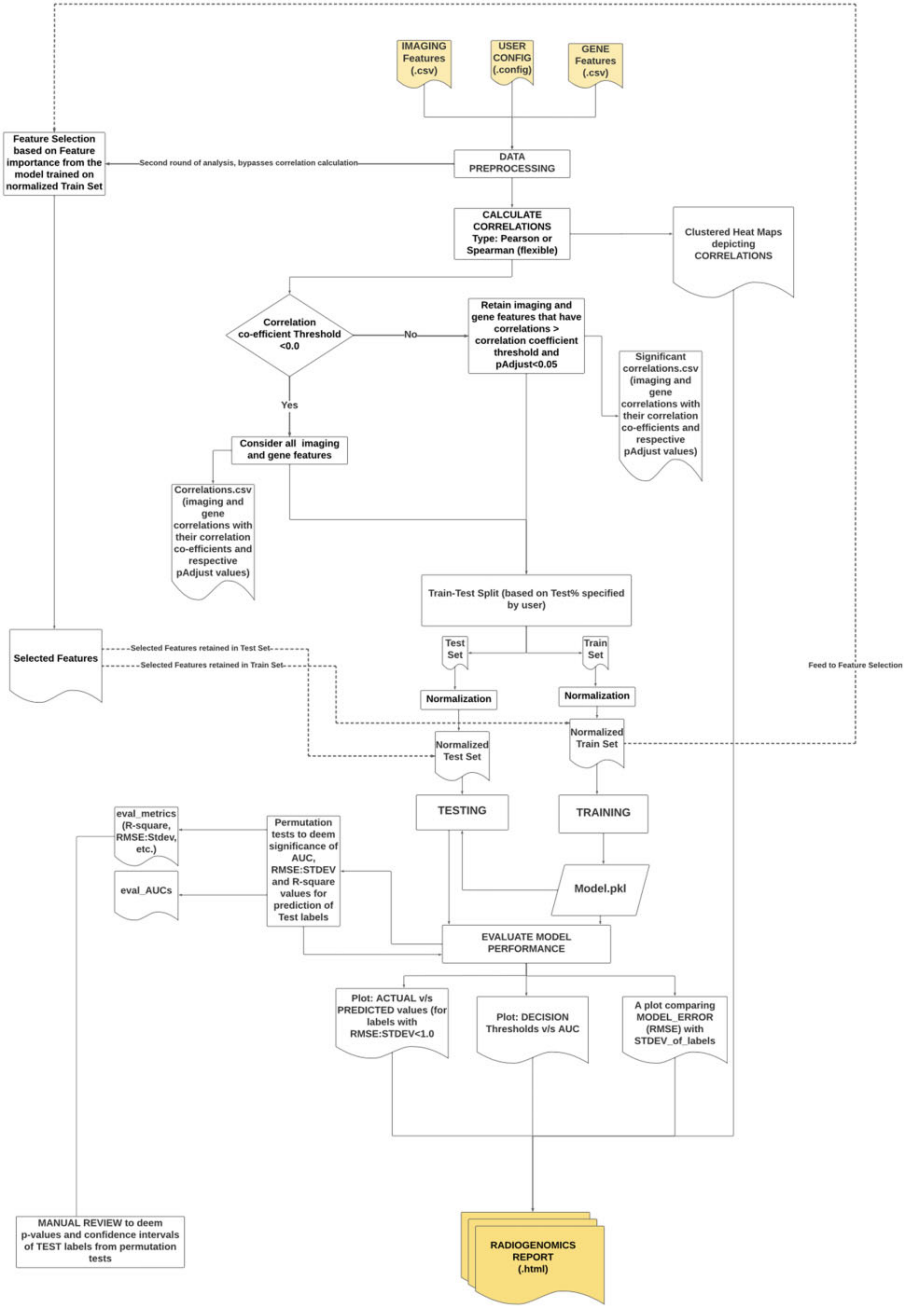
ImaGene Workflow: calculating correlations, conducting feature selections and training and testing of radiogenomic machine learning models

In addition, ImaGene executes a second round of radiogenomic analysis using features purely selected by the model using either feature importances or model coefficients from the training of the respective model type using a normalized Train Set using ‘SelectFromModel’ functionality from scikit-learn library (Pedregosa *et al.*, 2011), and further training model based on such selected features only (Fig. 2). The correlation module is completely bypassed in this round and the results from the feature selection and the reports from model’s training, testing and permutation tests thereof are stored in a sub directory prefixed as ‘FeaturesSelFromModel’ within the downloadable results directory.

**Fig. 3.**
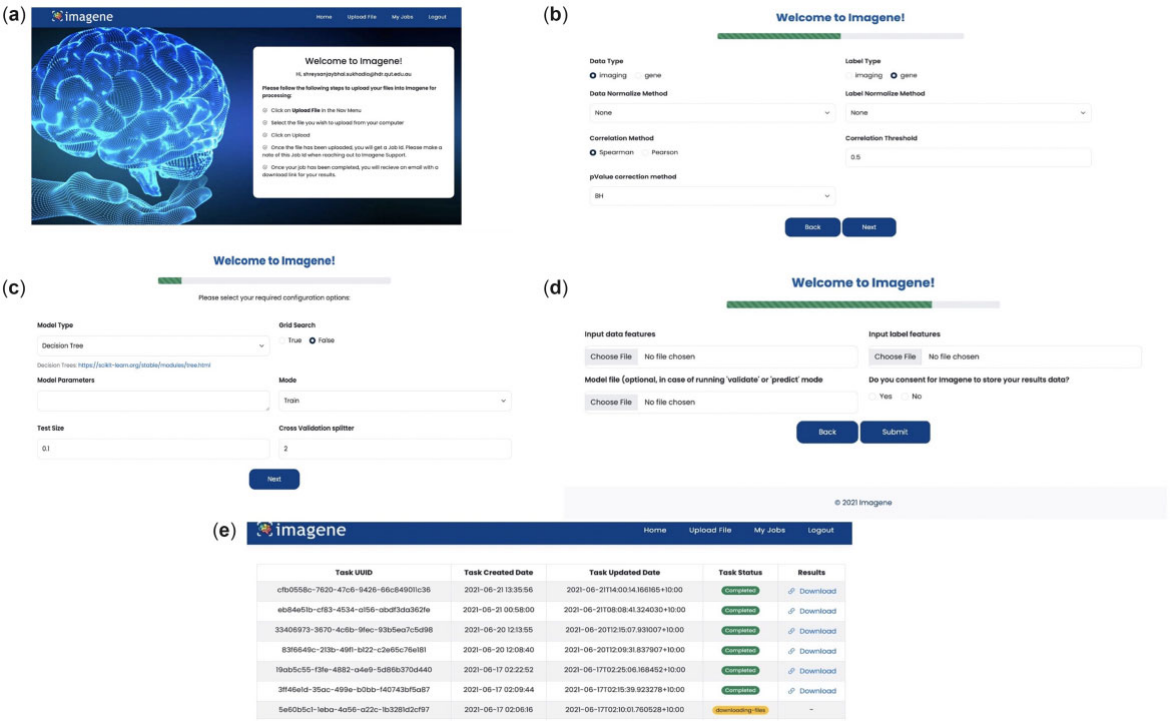
(**a**) Welcome page for ImaGene, (**b** and **c**) parameter selection for ImaGene, (**d**) selectingradiomic and genomic files, and model file depending on the mode of operation, and (**e**) job tracking and results download page

Validation of the model can be conducted using multiple test datasets in the validation mode (Fig. 3c). The prediction mode of the platform can be used to perform omics predictions on new imaging datasets (Fig. 3c). The ‘job tracking’ feature of ImaGene allows users to track the status of their experiments and to download the reports and the supporting result files (along with intermediate calculated tables) of completed experiments using the provided download links (Fig. 3e) on the ‘jobs’ page on the web platform.

### 2.2 Radiogenomics report

Once an experimental run is completed, users can use the job tracking page to download a detailed HTML report. This report represents the alterations made to the imaging and omics data in each module of ImaGene in the form of scores and plots which help users acquire an overview of the process and deduce meaningful outcomes from the features data (Supplementary Report BC 2). The first section of the report provides details regarding the type of input data (i.e. imaging and omics features files), the number of sample entries common to both files and the type of normalization method used for the input data. The second section provides hierarchically clustered heat maps depicting statistical correlations between the imaging and omics features. Features having a correlation coefficient greater than the default threshold of 0.5 and an false discovery rate (FDR)-adjusted *P*-value greater than the cutoff value of 0.05 are used to train user-defined AI models and set hyperparameters including the *K*-fold cross-validation splitter (i.e. the ‘cv’ parameter). The next section describes the imaging and omics features that exhibited significant correlations and that were used to train the model along with the parameters that were used for training and testing the model. The next section is the ‘model interpretation’ section which provides metrics such as MSE, RMSE and *K*-fold cross-validation scores of the model, and various plots such as a bar plot representing the RMSE:STDEV ratio for each label feature, a scatter plot representing true versus predicted values of labels having RMSE:STDEV ≤ 1, and a two-dimensional plot representing dt values on the *x*-axis and AUC values on the *y*-axis to indicate the performance of the model in classifying labels (genes) into categories (high or low expression) at different cutoff values (normalized gene expressions—FPKMs) from 0 to 1. These metrices aid users determine the model’s reliability in predicting and/or classifying labels. Furthermore, the reports also include similar metrices and plots from permutation tests on gene labels in the ‘validation permuts’ sections therein.

## 3 Results

To demonstrate the performance and capabilities of ImaGene, we conducted two case study analyses of tumor imaging features already published in TCIA and corresponding gene expression data from TCGA pertaining to two major types of cancer—invasive breast carcinoma (IBC) and head and neck squamous cell carcinoma (HNSCC) (Burnside *et al.*, 2016; Clark *et al.*, 2013; Li *et al.*, 2016a, b; Zhu *et al.*, 2015). To clarify further, these cases do not necessarily obligate the user to use ImaGene’s parameters similar to ours, and users have the flexibility to operate on the same datasets using parameters of their choice. The imaging (or radiomic) and gene expression datasets are provided as supplementary tables for IBC and HNSCC (Supplementary Table BC Gene, Supplementary Table BC Radiomic features, Supplementary Table HNSCC Gene and Supplementary Table HNSCC Radiomic features).

### 3.1 Case study 1: IBC

The TCGA Breast Phenotype Research Group datasets available on the TCIA platform were accessed to download 36 imaging features of tumor ROIs in MRI scans of 89 IBC patients already published by expert group of radiologists on the TCIA platform (Burnside *et al.*, 2016; Clark *et al.*, 2013; Li *et al.*, 2016a, b; Zhu *et al.*, 2015). A gene expression dataset comprised of FPKM (Fragments Per Kilobase of transcript per Million mapped reads) data was downloaded from the main TCGA-BRCA sample set of which the 89 patient samples formed a subset. FPKM data was screened for genes that had an FPKM value greater than or equal to 5 for at least 30 patients in the TCGA-BRCA cohort. In total, 976 genes were obtained that met with these criteria. This type of screening ensures reliable correlation-detection between imaging and omics data (gene-FPKM data in this case study) such that these results can be leveraged for the generation of high-quality AI models capable of generating predictions or classifying gene expression levels based upon tumor ROI imaging features.

Once the initial screening of the downloaded dataset was complete, the imaging and gene-FPKM features were processed based upon user-defined ImaGene parameter settings. For example, the absolute Pearson-based correlation coefficient threshold was set to 0.7 for linear regression (LR) modeling downstream and 0.6 for other model types depending on number of features required for the satisfactory training of different types of AI models, the *P*-value correction method for correlations was set to BH with the corrected *P*-value threshold set to 0.05 by default. Furthermore, the imaging features were set as data and gene-FPKM values were set as labels. For both data and labels, the normalization method was set to StandScaler, the test size was set to 0.2 (i.e. 20% of the dataset) and the *K*-fold cross-validation splitter (cv) was set to 2 for LR and DT models, and 3 for other model types.

At the end of the experimental run using the above mentioned parameters, a comprehensive radiogenomics report was obtained for each model type that reported LR, DT, Multi-Task LASSO (MTL) and Multi-Task Elastic Net (MTEN) models (Supplementary Reports BC 1-5) that yielded the best results for the test dataset. Each model was generated twice in a fully automated fashion, i.e. executing two scenarios: one with and another without a prior correlation-threshold-based filtering of features in a single run attempt, with the latter including an additional feature selection step (i.e. filtering features based on feature importances or correlation weights yield by the respective model type, i.e. DT or purely regression models) prior to training and testing of the models to ultimately improve gene predictions by them. Note that the correlation filtering in the first scenario occurred on a nonnormalized dataset. Normalization occurred for the train and test datasets independent of each other, i.e. post-train-test split, depending on the normalization method selected by the user (Table 1). The radiogenomic reports from these runs highlighted four radiomics features—(i) signal enhancement ratio (K7), (ii) size of the lesion/volume (S1), (iii) surface area (S3) and (iv) volume of the most enhancing voxels (S4)—that were first significantly correlated (FDR-adjusted *P*-value, aka *P*-adjust <0.05) with the FPKM values of a total of 17 genes as depicted in the reports (Supplementary Report LR CorrF BC1), i.e. when the correlation coefficient threshold was set to 0.7. Second, when the correlation coefficient threshold was relaxed to 0.6, an additional radiomics feature (Washout rate (K4)) and 15 additional genes appeared on the list of features exhibiting significant correlations (Supplementary Report DT CorrF BC2). Using this threshold and the expanded feature set, the training of the DT, MTL and MTEN models was found to be enhanced but none identified significant genes (*P* < 0.05) posttest with the test dataset. LR identified one significant gene posttest-SLC22A31 (Table 2). On contrary, when these models got trained with the features selected from the feature selection module (using feature importances or correlation weights yield by the respective model type) rather than correlation coefficient threshold-based feature-filtering, they identified eight more significant genes (*P* < 0.05) with at least one gene predicted by each model type (Supplementary Report LR FS BC3, Supplementary Report DT FS BC4, Supplementary Report MTL FS BC5, Supplementary Report MTEN FS BC6, and Table 2). Note that in order to establish significance of the predicted genes, ImaGene conducts permutation tests for all the genes detected at AUC > 0.9 and *R*^2^ > 0.25 during the test phase and the *P*-values are calculated using the results thereof which ultimately helps reduce the list of genes to the significant ones only (i.e. *n* = 9 in this case).

The biological significances of the identified genes were ascertained using existing literature. Wilm’s tumor gene 1 (WT1) is oncogenic as well as a tumor suppressor in breast cancer. The high expression of WT1 regulates proliferation, migration and apoptosis of breast cancer cells by interacting with molecules or signaling pathways such as the Caspase family, EphA2, p53, HER2, TP-alpha and ER-alpha (Zhang *et al.*, 2020). WT1 vaccines against breast cancer and other types of cancer such as lung, leukemia and pancreatic cancer are currently under phase I or phase II clinical trials highlighting its value in precision medicine (Zhang *et al.*, 2020). Leucine-rich glioma inactivated 3 (LGI3) transduces signals through proteins implicated in cancer and its higher expression correlates with higher survival rates indicating its tumor suppressive nature in cancer (Kwon *et al.*, 2018). Orosomucoid 1 (ORM1) upregulates the expression of matrix metalloproteinases (MMP-2 and MMP-9) consequently promoting epirubicin resistance in breast cancer (Qiong and Yin, 2021). A loss of expression of DSG1 has been significantly associated with better cancer-specific survival in anal squamous cell carcinoma (SCC) which encourages us to study the role of DSG1 in breast cancer given its wide range of fpkm expression, i.e. 0.4–0.7 (low to high) detected at AUC of 0.94 by ImaGene (Myklebust *et al.*, 2012). Claudin 10 (CLDN10) has been found to be associated with ovarian cancer progression via TGF-beta or WNT/Beta-catenin induced epithelial to mesenchymal transition (Li *et al.*, 2020). Smoothen Like 2 (SMTNL2) is downregulated in breast cancer (Gálvez-Santisteban *et al.*, 2012). Cystatin-SN’s (CST1) high expression promotes breast cancer and predicts poor prognosis in patients (Dai *et al.*, 2017). Solute Carrier Family 22 Member 31 (SLC22A31) was identified as one of the prognostic genes in right-sided colon cancer (Liang *et al.*, 2018), which warrants exploration of its significance in other cancer types as well, including the breast cancer. Such biological evidence from the literature that matches with the evidence from ImaGene encourages us to explore the role of these 9 genes as targetable, therapeutic and monitoring radiogenomic biomarkers in IBC.

In summary, ImaGene facilitates the systematic and user-controlled imaging-based prediction and classification of gene expression in IBC, demonstrating the capability of this platform to identify significant associations between imaging and omics features using correlation analysis as well as AI models in an algorithmic manner. It also allows complete control over multiple operational parameters, ultimately producing a transparent radiogenomics report along with all supporting information outcomes necessary for user interpretation.

### 3.2 Case study 2: HNSCC

The TCGA HNSCC cohort was accessed to download 540 radiomics features from CT scans of 106 HNSCC patients from a previous imaging study conducted at Stanford University (Mukherjee *et al.*, 2020). Consistent with the results of case study 1, the gene expression data (FPKM files) for the entire cohort were downloaded and genes that exhibited FPKM values of greater than or equal to 5 in at least 30 patients were selected for further processing.

The radiomics and gene-FPKM features from 106 samples were used for the experimental run along with the following parameter settings: (i) the absolute Pearson-based correlation coefficient threshold was set to 0.7 for LR modeling and 0.6 for other model types and (ii) the *P*-value correction method was set to BH with the corrected *P*-value threshold set to 0.05 by default. Radiomics features were set as data and gene-FPKM values were set as labels. The feature normalization method was set to StandScaler for both inputs, the test size was set to 0.2 (i.e. 20% of the dataset), and the *K*-fold cross-validation splitter (cv) was set to 3.

At the end of the experimental run, a comprehensive radiogenomics report was obtained for each model type (Supplementary Report HNSCC 1-6). These reports presented significant correlations between 12 radiomics features (pertaining to texture, shape, size and wavelet category) and FPKM values for 21 genes (Supplementary Report LR CorrF HNSCC1). When the correlation coefficient threshold was relaxed to 0.6, 29 additional radiomics features in the wavelet category and 33 additional genes appeared on the list of significant correlations. This expanded feature set was found to improve the training of the DT model (Supplementary Report DT CorrF HNSCC2), although it did not yield any significant (*P* < 0.05) genes posttest unlike LR that did yield a significant (*P* < 0.05) gene: MAL posttest (Table 3). On contrary, all the models (i.e. LR, DT, MTL and MTEN) were trained with the features selected from the feature selection module (using feature importances or correlation weights yield by the respective model type) rather than correlation coefficient threshold-based feature-filtering, they identified seven more significant genes (*P* < 0.05) with at least one gene predicted by three model types (i.e. DT, MTL and MTEN), none predicted by LR (Supplementary Report DT FS HNSCC4, Supplementary Report MTL FS HNSCC5, Supplementary Report MTEN FS HNSCC6 and Supplementary Report LR FS HNSCC3, and Table 3).

The biological significances of the identified genes were explored within existing literature. Nuclear Receptor Subfamily 0 Group B Member 1 (NR0B1) was involved in the resistance against anti-cancer drugs and invasion of lung adenocarcinoma cell lines (Oda *et al.*, 2009). A high expression of NR0B1 has shown high levels of tumor metastasis (Oda *et al.*, 2009). Augmented with the strong evidence from ImaGene (Table 3), and as evidence by literature, investigating the role of NR0B1 in HNSCC and other cancer types further will be important. An overexpression of the group A phospholipase A2 (PLA2G2A) dictates poor prognostic outcomes in rectal cancer (He *et al.*, 2015). Increased expression of PL2G2A was cited in human oral squamous cell carcinoma (OSCC) and mouse skin cancer tissues (Chovatiya *et al.*, 2019). PL2G2A knockdowns in OSCC and skin SCC showed a coreduction of tumor volume in nonobese diabetic/severe combined immunodeficient mice, and in the signaling of c-Jun N-terminal Kinase (Chovatiya *et al.*, 2019). All these findings combined with strong evidence from ImaGene warrant the exploration of PL2G2A’s impact in other SCCs including HNSCC type. The epigenetic inactivation MAL, a tumor suppressor, is also a crucial biomarker in HNSCC (Cao *et al.*, 2010). The exogenous overexpression and cell-death induction by MAL gene transfer (Cao *et al.*, 2010) highlight its therapeutic capabilities in HNSCC. Such findings of the strong association of MAL overexpression, along with radiology imaging features from ImaGene, highlight the strength of ImaGene to conduct noninvasive radiogenomic studies in HNSCC.

Claudin-16 (CLDN16) is a part of the seven-gene signature that predicts prognosis in OSCC (Ribeiro *et al.*, 2021). Elevated levels of PRDM14 in human primordial germ-like cells (PGCLCs) are known to impart defects at proliferation and differentiation stages in germline (Gell *et al.*, 2018). Vertnin (VRTN) is a vital transcription factor in the development of thorax, i.e. neck-to-abdomen anatomy in mammals (Duan *et al.*, 2018). VRTN could modulate somite segmentation interacting with notch signaling pathways (Duan *et al.*, 2018). ImaGene does show significant oscillating levels of VRTN expressions in HNSCC patients (Table 3) which could be indicative of VRTN’s role in modulating HNSCC consequently making VRTN a good candidate for further research in HNSCC. Leucine-rich Repeat Neuronal 1 (LRRN1) was studied in neuroepithelial cells in chicks and found to be regulating components involved in cell-cell recognition pathways (Andreae *et al.*, 2007). LRRN’s protein domains were found to be conserved across chick, mouse and human proteome—indicative of their similar regulatory role in humans (Andreae *et al.*, 2007). A significant detection of LRNN’s high expression levels in HNSCC patients by ImaGene (Table 3) combined with the citations in literature does warrant an investigation of LRNN’s role in HNSCC. MDS1 and EVI1 complex locus (MECOM) serves as one of the crucial prognostic markers handling immune cell responses in lung adenocarcinoma (Li *et al.*, 2022). EV1 has been shown to promote cellular proliferation and invasiveness in HNSCC (Grandits *et al*., 2022). The strong evidence from literature, along with a significant detection of MECOM’s high expression levels by ImaGene, warrants further investigation of MECOM in HNSCC.

Overall, these two case studies highlight many important genes that may contribute to IBC and HNSCC incidence and/or progression, thus illustrating the capability of ImaGene as a tool for identifying meaningful associations between imaging features and gene expression data in tumor patients using correlation analysis or feature selections. When used in conjunction with AI modeling techniques, ImaGene is a meaningful resource that can enable valuable tumor radiogenomic evaluation and reporting.

## 4 Discussion

ImaGene is a web-based platform that offers a comprehensive radiogenomic framework to perform statistical correlations and AI modeling techniques as a means of linking molecular and tumor imaging data. It enables the rapid screening of significantly correlated radiographic and omics features for particular tumors, as well as the construction and testing of different types of AI models using these features as a means of aiding in the prediction of biologically relevant omics features based upon imaging findings. This platform provides control over radiogenomic experiments by hosting multiple configuration parameters that can be defined by end users or left at default values. It also obeys FAIR principles (Wilkinson *et al.*, 2016), allowing users to safely store and track their experimental data files and to download reports using links available on the job tracking page. The reports are comprehensive HTML documents that provide output from each step of the algorithm, making it easy for users to track the processing of the imaging and omics features through statistical tests and AI modeling. These reports highlight the transparency and easy interpretability of the AI models. The hierarchically clustered heat maps given in these reports represent univariate, bivariate and multivariate statistical correlations between the imaging and omics features, and the MSE, RMSE:STDEV and AUC values reflect the reliability of the AI models when predicting and classifying omics labels from imaging data. Thus, these reports prepared by the platform provide a comprehensive overview of the entire analytical process and permit users to interpret their datasets while providing with the experimental parameters necessary to arrive at meaningful conclusions.

ImaGene aims to advance current AI modeling techniques through its flexibility, ease of interpretation and user-friendly design, ensuring that users can confidently design and interpret the radiogenomic experiments in their omic research laboratories with accessible data from their partner radiological laboratories (or vice versa). Relative to other radiogenomic applications such as Imaging-Amaretto, Imaging-Community Amaretto and Musa, ImaGene’s features have the potential to make it widely adoptable in clinical settings and laboratories worldwide as a tool for testing associations between imaging and omics data for all tumor types, paving the way for significant discoveries in the fields of radiogenomics and oncological research.

As a proof of concept, we tested the performance of ImaGene for the prediction and classification of omics profiles from imaging features using the IBC and HNSCC datasets. We identified strong associations between the expression of genes WT1, LGI3, SP7, DSG1, ORM1, CLDN10, CST1, SMTNL2 and SLC22A31, and their imaging features such as tumor size, shape, enhancement and kinetic curve assessments in the IBC dataset (Supplementary Report LR CorrF BC1, Supplementary Report DT CorrF BC2, Supplementary Report LR FS BC3, Supplementary Report MTL FS BC5, Supplementary Report MTEN FS BC6, and Table 2). Further research into the roles of these genes in cancer revealed that they either have considerable evidence or a strong basis for playing an indirect or direct role in shaping IBC tumorigenesis. These findings illustrate the need for further research in IBC cancer type. In our HNSCC case study, ImaGene further detected strong associations between the expression of eight genes (i.e. NR0B1, PLA2G2A, MAL, CLDN16, PRDM14, VRTN, LRRN1 and MECOM) and imaging attributes such as tumor texture, shape, size and wavelet features (Supplementary Report LR CorrF HNSCC1, Supplementary Report DT CorrF HNSCC2, Supplementary Report LR FS HNSCC3, Supplementary Report DT FS HNSCC4, Supplementary Report MTL FS HNSCC5, Supplementary Report MTEN FS HNSCC6, and Table 3). Further research into the roles of these genes in cancer revealed that they either have considerable evidence or a strong basis of playing an indirect or direct role in shaping HNSCC tumorigenesis, thus warranting further research in HNSCC cancer type.

ImaGene can facilitate collaborations between researchers, making the sharing of radiogenomics reports more convenient and ultimately leading to the advancement of research in the cancer and precision medicine space. Our case studies have sought to provide a basis for the radiogenomic analysis through the processing of imaging and omics feature data in two tumor types while permitting robust control over experimental parameters. We have successfully demonstrated the configuration and usage of statistical correlations and AI modeling on our platform, leading to the construction of reliable AI models that can predict biologically relevant omic features from imaging data. Analysis of multiple datasets of different types and subtypes of tumors in ImaGene can contribute to the development of a radiogenomic knowledge base that can be used in cancer research, drug discovery and precision medicine, allowing the outcomes of this platform to undergo translation for clinical use in the near future.

## Data Availability

The data underlying this article are available in the article and in its online supplementary material, i.e., https://github.com/skr1/Imagene.git.

## References

[vbac079-B1] Aihara M. et al (1994) Heterogeneity of prostate cancer in radical prostatectomy specimens. Urology, 43, 60–66. 8284886 10.1016/s0090-4295(94)80264-5

[vbac079-B2] Andreae L.C. et al (2007) Analysis of Lrrn1 expression and its relationship to neuromeric boundaries during chick neural development. Neural Dev., 2, 22–22. 17973992 10.1186/1749-8104-2-22 PMC2225406

[vbac079-B3] Anker J. et al (2015) Genomic landscape of DNA repair genes in cancer: mutation and copy number variation (CNV) frequencies. J. Clin. Oncol., 33, 2557–2557.

[vbac079-B4] Ashraf A.B. et al (2014) Identification of intrinsic imaging phenotypes for breast cancer tumors: preliminary associations with gene expression profiles. Radiology, 272, 374–384. 24702725 10.1148/radiol.14131375 PMC4564060

[vbac079-B5] Attiyeh M.A. et al (2019) CT radiomics associations with genotype and stromal content in pancreatic ductal adenocarcinoma. Abdom. Radiol., 44, 3148–3157. 10.1007/s00261-019-02112-1 PMC6692205 31243486

[vbac079-B6] Bakr S. et al (2018) A radiogenomic dataset of non-small cell lung cancer. Sci. Data, 5, 180202–180204. 30325352 10.1038/sdata.2018.202 PMC6190740

[vbac079-B7] Balassiano K. et al (2011) Aberrant DNA methylation of cancer-associated genes in gastric cancer in the European Prospective Investigation into Cancer and Nutrition (EPIC–EURGAST). Cancer Letters, 311, 85–95. 21831520 10.1016/j.canlet.2011.06.038

[vbac079-B8] Bodalal Z. *et al.* (2019). Radiogenomics: bridging imaging and genomics. Abdom. Radiol., 44, 1960–84. 10.1007/s00261-019-02028-w 31049614

[vbac079-B9] Bosaily AEl-Shater . (2016) The concordance between the volume hotspot and the grade hotspot: a 3-D reconstructive model using the pathology outputs from the PROMIS trial. Prostate Cancer Prostatic Dis., 19, 322–344. 27502740 10.1038/pcan.2016.37 PMC5411669

[vbac079-B10] Boutros P.C. et al (2015) Spatial genomic heterogeneity within localized, multifocal prostate cancer. Nat. Genet., 47, 736–745. 26005866 10.1038/ng.3315

[vbac079-B1000] Brito J.J. et al (2020) Corrigendum to: Recommendations to enhance rigor and reproducibility in biomedical research. Gigascience, 9, giaa103. 10.1093/gigascience/giaa103 PMC7495904 32940333

[vbac079-B11] Burnside E.S. et al (2016) Using computer-extracted image phenotypes from tumors on breast MRI to predict breast cancer pathologic stage. Cancer, 122, 748–757. 26619259 10.1002/cncr.29791 PMC4764425

[vbac079-B12] Cao W. et al (2010) Epigenetic silencing of MAL, a putative tumor suppressor gene, can contribute to human epithelium cell carcinoma. Mol. Cancer, 9, 296–296. 21092172 10.1186/1476-4598-9-296 PMC3002926

[vbac079-B13] Chen M. et al (2013) Identification of human HK genes and gene expression regulation study in cancer from transcriptomics data analysis. PLoS One, 8, e54082. 23382867 10.1371/journal.pone.0054082 PMC3561342

[vbac079-B14] Chitalia R.D. et al (2020) Imaging phenotypes of breast cancer heterogeneity in preoperative breast dynamic contrast enhanced magnetic resonance imaging (DCE-MRI) scans predict 10-Year recurrence. Clin. Cancer Res., 26, 862–869. 31732521 10.1158/1078-0432.CCR-18-4067 PMC7024654

[vbac079-B15] Chovatiya G.L. et al (2019) Context-dependent effect of sPLA2-IIA induced proliferation on murine hair follicle stem cells and human epithelial cancer. eBioMedicine, 48, 364–376. 31521610 10.1016/j.ebiom.2019.08.053 PMC6838435

[vbac079-B16] Clark K. et al (2013) The Cancer Imaging Archive (TCIA): maintaining and operating a public information repository. J. Digit. Imaging, 26, 1045–1057. 23884657 10.1007/s10278-013-9622-7 PMC3824915

[vbac079-B17] Colen R. et al (2014) NCI workshop report: clinical and computational requirements for correlating imaging phenotypes with genomics signatures. Transl. Oncol., 7, 556–569. 25389451 10.1016/j.tranon.2014.07.007 PMC4225695

[vbac079-B18] Dai D.-N. et al (2017) Elevated expression of CST1 promotes breast cancer progression and predicts a poor prognosis. J. Mol. Med., 95, 873–886. 28523467 10.1007/s00109-017-1537-1 PMC5515997

[vbac079-B19] Depeursinge A. et al (2018). Locoregional Radiogenomic Models Capture Gene Expression Heterogeneity in Glioblastoma. Cold Spring Harbor Laboratory Press, Cold Spring Harbor.

[vbac079-B20] Diaz O. et al (2021) Data preparation for artificial intelligence in medical imaging: a comprehensive guide to open-access platforms and tools. Phys. Med., 83, 25–37. 33684723 10.1016/j.ejmp.2021.02.007

[vbac079-B21] Duan Y. et al (2018) VRTN is required for the development of thoracic vertebrae in mammals. Int. J. Biol. Sci., 14, 667–681. 29904281 10.7150/ijbs.23815 PMC6001657

[vbac079-B22] Freymann J.B. et al (2012) Image data sharing for biomedical research-meeting HIPAA requirements for de-identification. J. Digit. Imaging, 25, 14–24. 22038512 10.1007/s10278-011-9422-x PMC3264712

[vbac079-B23] Gálvez-Santisteban M. et al (2012) Synaptotagmin-like proteins control the formation of a single apical membrane domain in epithelial cells. Nat. Cell Biol., 14, 838–849. 22820376 10.1038/ncb2541 PMC3433678

[vbac079-B24] Gell J.J. et al (2018) PRDM14 is expressed in germ cell tumors with constitutive overexpression altering human germline differentiation and proliferation. Stem Cell Res., 27, 46–56. 29324254 10.1016/j.scr.2017.12.016 PMC5858915

[vbac079-B25] Gevaert O. et al (2012) Non-small cell lung cancer: identifying prognostic imaging biomarkers by leveraging public gene expression microarray data—methods and preliminary results. Radiology, 264, 387–396. 22723499 10.1148/radiol.12111607 PMC3401348

[vbac079-B26] Gevaert O. et al (2014) Glioblastoma multiforme: exploratory radiogenomic analysis by using quantitative image features. Radiology, 273, 168–174. 24827998 10.1148/radiol.14131731 PMC4263772

[vbac079-B27] Gevaert O. et al (2020) Imaging-AMARETTO: an imaging genomics software tool to interrogate multiomics networks for relevance to radiography and histopathology imaging biomarkers of clinical outcomes. JCO Clin. Cancer Inform., 4, 421–435. 32383980 10.1200/CCI.19.00125 PMC7265792

[vbac079-B28] Gillies R.J. et al (2016) Radiomics: images are more than pictures, they are data. Radiology, 278, 563–577. 26579733 10.1148/radiol.2015151169 PMC4734157

[vbac079-B29] González-Reymúndez A. , Vázquez A.I. (2020) Multi-omic signatures identify pan-cancer classes of tumors beyond tissue of origin. Sci. Rep., 10, 8341–8382. 32433524 10.1038/s41598-020-65119-5 PMC7239905

[vbac079-B30] Grandits A.M. et al (2022) EVI1 promotes the proliferation and invasive properties of human head and neck squamous cell carcinoma cells. Int. J. Mol. Sci., 23, 1050. 10.3390/ijms23031050 PMC8835242 35162973

[vbac079-B31] van Griethuysen J.J.M. et al (2017) Computational radiomics system to decode the radiographic phenotype. Cancer Res., 77, e104–e107. 29092951 10.1158/0008-5472.CAN-17-0339 PMC5672828

[vbac079-B3000] Lee H.Y. et al (2013) Histopathology of lung adenocarcinoma based on new IASLC/ATS/ERS classification: Prognostic stratification with functional and metabolic imaging biomarkers. J. Magn. Reson. Imaging, 38, 905–913. 23908132 10.1002/jmri.24080

[vbac079-B32] Lo Gullo R. et al (2020) Combining molecular and imaging metrics in cancer: radiogenomics. Insights Imaging, 11, 1–17. 31901171 10.1186/s13244-019-0795-6 PMC6942081

[vbac079-B33] He H.-L. et al (2015) PLA2G2A overexpression is associated with poor therapeutic response and inferior outcome in rectal cancer patients receiving neoadjuvant concurrent chemoradiotherapy. Histopathology, 66, 991–1002. 25393083 10.1111/his.12613

[vbac079-B34] Incoronato M. et al (2020) Correlating imaging parameters with molecular data: an integrated approach to improve the management of breast cancer patients. Int. J. Biol. Markers, 35, 47–50. 10.1177/1724600819899665 32079469

[vbac079-B2000] Kamel H.F.M. , Al-Amodi H. (2017) Exploitation of gene expression and cancer biomarkers in paving the path to era of personalized medicine. Genomics Proteomics Bioinformatics, 15, 220–235. 28813639 10.1016/j.gpb.2016.11.005 PMC5582794

[vbac079-B35] Koçak B. et al (2019) Radiomics with artificial intelligence: a practical guide for beginners. Diagn. Interv. Radiol., 25, 485–495. 31650960 10.5152/dir.2019.19321 PMC6837295

[vbac079-B36] Kvale R. (2009) Concordance between Gleason scores of needle biopsies and radical prostatectomy specimens: a population-based study. BJU Int., 103, 1647–1654. 19154461 10.1111/j.1464-410X.2008.08255.x

[vbac079-B37] Kwon N.S. et al (2018) Leucine-rich glioma inactivated 3: integrative analyses reveal its potential prognostic role in cancer. Mol. Med. Rep., 17, 3993–4002. 29257304 10.3892/mmr.2017.8279

[vbac079-B38] Li H. et al (2016a) MR imaging radiomics signatures for predicting the risk of breast cancer recurrence as given by research versions of MammaPrint, oncotype DX, and PAM50 gene assays. Radiology, 281, 382–391. 27144536 10.1148/radiol.2016152110 PMC5069147

[vbac079-B39] Li H. et al (2016b) Quantitative MRI radiomics in the prediction of molecular classifications of breast cancer subtypes in the TCGA/TCIA data set. NPJ Breast Cancer, 2, 16012–16012. 27853751 10.1038/npjbcancer.2016.12 PMC5108580

[vbac079-B40] Li M. et al (2022) MECOM/PRDM3 and PRDM16 serve as prognostic-related biomarkers and are correlated with immune cell infiltration in lung adenocarcinoma. Front. Oncol., 12, 772686–772612. 35174083 10.3389/fonc.2022.772686 PMC8841357

[vbac079-B41] Li Z. et al (2020) Claudin 10 acts as a novel biomarker for the prognosis of patients with ovarian cancer. Oncol. Lett., 20, 373–381. 10.3892/ol.2020.11557 PMC7285858 32565963

[vbac079-B42] Liang L. et al (2018) Distinguishable prognostic signatures of left- and right-sided colon cancer: a study based on sequencing data. Cell. Physiol. Biochem., 48, 475–490. 30016783 10.1159/000491778

[vbac079-B44] Martin-Gonzalez P. et al (2020) Integrative radiogenomics for virtual biopsy and treatment monitoring in ovarian cancer. Insights Imaging., 11, 94–94. 32804260 10.1186/s13244-020-00895-2 PMC7431480

[vbac079-B4000] Mobadersany P. et al (2018) Predicting cancer outcomes from histology and genomics using convolutional networks. Proc. Natl. Acad. Sci. U S A, 115, E2970–E2979. 29531073 10.1073/pnas.1717139115 PMC5879673

[vbac079-B45] Mukherjee P. et al (2020) CT-based radiomic signatures for predicting histopathologic features in head and neck squamous cell carcinoma. Radiol. Imaging Cancer, 2, 190039–190078. 10.1148/rycan.2020190039 PMC7263288 32550599

[vbac079-B46] Myklebust M.P. et al (2012) Expression of DSG1 and DSC1 are prognostic markers in anal carcinoma patients. Br. J. Cancer, 106, 756–762. 22333708 10.1038/bjc.2011.548 PMC3322941

[vbac079-B47] Nasrallah M.P. et al (2019) Molecular neuropathology in practice: clinical profiling and integrative analysis of molecular alterations in glioblastoma. Acad. Pathol., 6, 2374289519848353. 31206012 10.1177/2374289519848353 PMC6537274

[vbac079-B48] Oda T. et al (2009) Tumorigenic role of orphan nuclear receptor NR0B1 in lung adenocarcinoma. Am. J. Pathol., 175, 1235–1245. 19644015 10.2353/ajpath.2009.090010 PMC2731142

[vbac079-B49] Park C.-H. et al (2010) Identification of novel gastric cancer-associated CNVs by integrated analysis of microarray. J. Surg. Oncol., 102, 454–461. 20872948 10.1002/jso.21585

[vbac079-B50] Pedregosa F. et al (2011) scikit-learn: machine learning in Python. J. Mach. Learning Res., 12, 2825–2855.

[vbac079-B51] Peng L. et al (2015) Large-scale RNA-Seq transcriptome analysis of 4043 cancers and 548 normal tissue controls across 12 TCGA cancer types. Sci. Rep., 5, 13413–13426. 26292924 10.1038/srep13413 PMC4544034

[vbac079-B52] Pfaehler E. et al (2019) RaCaT: an open source and easy to use radiomics calculator tool. PLoS One, 14, e0212223. 30785937 10.1371/journal.pone.0212223 PMC6382170

[vbac079-B53] Prior F. et al (2017) The public cancer radiology imaging collections of The Cancer Imaging Archive. Sci. Data, 4, 170124. 28925987 10.1038/sdata.2017.124 PMC5827108

[vbac079-B54] Qiong L. , Yin J. (2021) Orosomucoid 1 promotes epirubicin resistance in breast cancer by upregulating the expression of matrix metalloproteinases 2 and 9. Bioengineered, 12, 8822–8832. 34654351 10.1080/21655979.2021.1987067 PMC8806942

[vbac079-B5000] Rice T.W. et al (2017) 8th edition AJCC/UICC staging of cancers of the esophagus and esophagogastric junction: Application to clinical practice. Ann. Cardiothorac. Surg., 6, 119–130. 28447000 10.21037/acs.2017.03.14 PMC5387145

[vbac079-B55] Ribeiro I.P. et al (2021) A seven-gene signature to predict the prognosis of oral squamous cell carcinoma. Oncogene, 40, 3859–3869. 33972685 10.1038/s41388-021-01806-5

[vbac079-B6000] Shukla G. et al (2017) Advanced magnetic resonance imaging in glioblastoma: a review. Chin. Clin. Oncol., 6, 40. 28841802 10.21037/cco.2017.06.28

[vbac079-B56] Siddiqui M.M. et al (2015) Comparison of MR/ultrasound fusion-guided biopsy with ultrasound-guided biopsy for the diagnosis of prostate cancer. J. Am. Med. Assoc., 313, 390–397. 10.1001/jama.2014.17942 PMC4572575 25626035

[vbac079-B57] Smith C.P. et al (2019) Radiomics and radiogenomics of prostate cancer. Abdom. Radiol., 44, pages2021–2029. 10.1007/s00261-018-1660-7 29926137

[vbac079-B58] Sottoriva A. et al (2013) Intratumor heterogeneity in human glioblastoma reflects cancer evolutionary dynamics. Proc. Natl. Acad. Sci. U. S. A., 110, 4009–4014. 23412337 10.1073/pnas.1219747110 PMC3593922

[vbac079-B59] Trivizakis E. et al (2020) Artificial intelligence radiogenomics for advancing precision and effectiveness in oncologic care (review). Int. J. Oncol., 57, 43–53. 32467997 10.3892/ijo.2020.5063 PMC7252460

[vbac079-B60] Wang W. et al (2018) Bioinformatic analysis of gene expression and methylation regulation in glioblastoma. J. Neurooncol., 136, 495–503. 29168084 10.1007/s11060-017-2688-1

[vbac079-B61] Wilkinson M.D. et al (2016) The FAIR guiding principles for scientific data management and stewardship. Sci. Data, 3, 160018–160036. 26978244 10.1038/sdata.2016.18 PMC4792175

[vbac079-B62] Zanfardino M. et al (2021) MuSA: a graphical user interface for multi-OMICs data integration in radiogenomic studies. Sci. Rep., 11, 1550–1550. 33452365 10.1038/s41598-021-81200-z PMC7811020

[vbac079-B63] Zhang Y. et al (2020) The role of WT1 in breast cancer: clinical implications, biological effects and molecular mechanism. Int. J. Biol. Sci., 16, 1474–1480. 32210734 10.7150/ijbs.39958 PMC7085227

[vbac079-B64] Zhou M. et al (2018) Non-small cell lung cancer radiogenomics map identifies relationships between molecular and imaging phenotypes with prognostic implications. Radiology, 286, 307–315. 28727543 10.1148/radiol.2017161845 PMC5749594

[vbac079-B65] Zhu Y. et al (2015) Deciphering genomic underpinnings of quantitative MRI-based radiomic phenotypes of invasive breast carcinoma. Sci. Rep., 5, 17787–17787. 26639025 10.1038/srep17787 PMC4671006

